# Peer Review of “COVID-19 Outcomes and Genomic Characterization of SARS-CoV-2 Isolated From Veterans in New England States: Retrospective Analysis”

**DOI:** 10.2196/35516

**Published:** 2021-12-17

**Authors:** Mathew Mbwogge

**Keywords:** infectious disease, COVID-19, epidemiology, veteran, outcome, sequencing, genetics, virus, United States, impact, testing, severity, mortality, cohort

*This is a peer-review report submitted for the paper “COVID-19 Outcomes and Genomic Characterization of SARS-CoV-2 Isolated From Veterans in New England States: Retrospective Analysis.”*

## Round 1 Review

### General Comments

The sudden menace imposed by the COVID-19 pandemic has led to the proliferation of studies on the epidemiology of viral genomics, specifically to understand disease risk factors, characteristics, and prognosis of those with COVID-19 [1-3]. Between 20% to 40% of COVID-19 admissions are reported to require intensive care [4], and have a fatality rate of 35% to 50% [5]. Many factors have been reported to either account for or to be associated with the clinical characteristics and prognosis of patients with COVID-19 [6-8]. Given that the aforementioned body of knowledge among veterans in New England is currently limited, the authors of the paper titled “COVID-19 Outcomes and Genomic Characterization of SARS-CoV-2 Isolated From Veterans in New England States” [9] investigated the patient characteristics, comorbidities, and disease predictors in a cohort of 426 veterans hospitalized for COVID-19. They found using a multivariate regression that age was the most significant predictor of being hospitalized, the severity of disease, and mortality; being non-White was more associated with being hospitalized; and those in need of oxygen upon admission were more likely to die.

Even though widely reported, genomic epidemiology remains a rapidly growing domain in virology [10]. Besides, the diversity of the four coronavirus genera (alpha, beta, gamma, and delta) [11] and the emergence and spreading of the B.1.1.7 variant from the United Kingdom, B.1.1.28 from Brazil, and B.1.351 from South Africa [12] warrant constant new data and knowledge translation. To this effect, this paper addresses a major area of concern and interest to the readership of the journal. The authors are clear in their title, which still needs to fully comply with the journal guidelines. The Abstract follows the guidelines and presents an overview of the study. Being an area that has received tremendous interest since the start of the COVID-19 pandemic, there was an overriding need for this study to be put in context. The paper’s introduction does well, ends with the study aim, and is brief at highlighting the main concern but deserves more attention. The general structure of the paper needs improvement to comply with the journal guidelines. The data collection methods, albeit needing clarification, seem reasonable with appropriate analysis, thereby giving value to the results. The discussion of the paper has been well articulated, and the conclusion ties with the research objective. The English used is simple and in plain language for easy comprehension.

Although congratulating the authors for a good attempt and concise paper, the paper will benefit from more value if the following specific comments are given consideration.

### Specific Comments

1. The general structure of the paper needs to conform to the journal guidelines.

2. The paper deserves to be put in context to be more appealing.

3. The introduction appears too restrictive and could be made more robust.

4. The methods and reported results warrant the use of appropriate guidelines.

5. All tables and figures need to be formatted following the guidelines.

6. Your references need slight improvement, in line with the guidelines.

To elucidate the aforementioned specific comments, kindly refer to the major and minor comments.

#### Major Comments

1. Kindly format your title following the guidelines [13]. Your title should normally end with a study design after a semicolon.

2. The methods subsection of the Abstract needs to summarize the study design; total sample, setting, and recruitment; mean age and gender differences; end points measured; data collection procedure; and data analysis. You may want to change the subtitle from “Study Design” to “Methods.”

3. Kindly use the following template to give your paper an overall structure that complies with the journal guidelines [14].

4. Given the high amount of reported literature in this field, I suggest putting your study in context [1]. Kindly search the Cochrane and Pubmed databases to:

1) Summarize the evidence already reported on the topic

2) Report why this study was necessary and the value added to the existing literature

3) The implication of all available evidence (including that from this study)

5. It will be good to structure your Introduction into Background, Study Rationale, and Study Aim.

6. Kindly structure your Methods section and report it as follows:

1) Specific objectives

2) Study design with justification (kindly make clear if this was a retrospective or prospective cohort study)

3) Study setting

4) Sample size calculations

5) Participant recruitment (with inclusion and exclusion criteria)

6) Sample/data collection

7) Sample handling procedure and quality control

8) Outcome measures (indicate whether these were continuous, binary, or categorical).

9) Whole genome sequencing and phylogenetic analysis

10) Data analysis (with justification for the approach used)

11) Ethical considerations

7. It is not clear whether this was a retrospective study since patients were still hospitalized at the time of this study. In 6.2 above, kindly be precise about the type of cohort study you undertook.

8. As part of your participant recruitment, indicate attempts made to reduce bias.

9. In 6.6 above, give details of those that collected samples and how that was done. If this was done by your research team, ensure to report the protocol used to collect samples. Organize your data collection into:

1) Hospitalization data

2) Peak disease severity data

3) Mortality data

4) Genome sequencing data

10. In 6.7 above, kindly clarify how samples were handled (including storage). If this was not done by the research team and was only reported, kindly indicate as such. If samples were not collected by you, provide details on how you had access to samples.

11. In 6.9 above, it is important to report the protocol/guidelines you used in genome sequencing. You may want to justify your procedure using these WHO guidelines [15] as well as substantiating your procedure with a visual display/flow of how the sequencing works.

12. As part of your statistical analysis, could you please justify your use of nonparametric tests? Kindly report the normality tests that were performed and the figures.

13. It might be worth arranging your data analysis first into univariate analysis and multivariate analysis, and then into hospitalization, peak disease severity, mortality, and genome sequencing.

14. In your data analysis, kindly report how you moved from univariate to multivariate analysis or how you selected variables for your multivariate model.

15. It is very important to indicate the guidelines used to report your review results. As part of your ethical considerations, indicate the guidelines you used to report your results. You may want to use these depending on which best suits your study method [16,17].

16. Your Results section should be reported in line with the Methods section starting with the participant characteristics. You might want to report your results as follows:

1) Participant characteristics

2) Predictors of hospitalization

3) Predictors of peak disease severity

4) Predictors of mortality

5) Genome sequencing and phylogenetics

17. Kindly move your Supplemental Table 1 to Participant Characteristics in the Results section.

18. Kindly move Supplemental Figure 1 and Supplemental Figure 2 to the Predictors of Hospitalization and Predictors of Mortality subsections of the Results section, respectively.

19. Note that the whole of your manuscript must be in portrait. You may want to highlight your Table 1 then click on “fit to window” on the automatic adjustment tab of Microsoft Word and move it together with Figure 1 to the Genomic Sequencing subsection of your Results section.

20. In the presentation of the results of your logistic regression, it will be good to state how the following assumptions were met:

1) Binary outcome

2) Linearity

3) Outliers

4) Multicollinearity

21. As part of the reported results of your regression, I suggest providing an explanation on your model’s goodness of fit by plotting and reporting the area under the receiver operating characteristic curve.

22. Kindly follow the guidelines to structure your Discussion section as follows:

1) Principal findings (summary)

2) Comparison with prior studies

3) Study limitations

23. Include a subsection “Author Contribution” after the Acknowledgments section to state the contribution of each author included in this paper.

24. Include a subsection “Conflicts of Interest” after “Author Contributions” to declare any conflict of interest.

25. Kindly list all Multimedia Appendices before the References section. For instance, your supplemental Table 2 will be labeled in the body of the manuscript as follows:

- Multimedia Appendix 1: Genomic lineage

26. Create a section “Abbreviations” after your references to list and expand all abbreviations in the text.

#### Minor Comments

27. You may want to include just the corresponding author on the manuscript and add all other authors in the metadata section of the online manuscript management system.

28. Kindly format your tables following the journal guidelines [18].

29. Kindly number your tables in the body of the text in order of appearance (Table 1, 2, 3, etc).

30. You need to report any *P* values based on the guidelines (eg, *P*=.05 or *P*<.001).

31. Review all your figures and their captions to ensure they are in line with the guidelines [19]. Apart from being uploaded as multimedia appendices, all figures must appear in the body of the text where they are first mentioned. The caption of each figure must appear at the bottom of the figure.

32. In your Discussion section, it will be appropriate to organize the “Comparison With Prior Studies” into subtitles as follows:

1) Predictors of hospitalization

2) Predictors of peak disease severity

3) Predictors of mortality

4) Genomic sequencing

33. I suggest starting your conclusion with a statement on the study objectives followed by a summary of findings, then lessons learned from your findings, and finally suggested direction of future research.

34. You need to delete your “Supplemental Table 2. Lineages of genomes” from the manuscript and upload it as a Multimedia Appendix in the online manuscript submission system. All multimedia appendices must be referenced in the body of your paper. Kindly have a look at other papers published in JMIRx Med.

35. Kindly make Acknowledgments, Funding, and Conflicts of Interest subsections.

36. Your references need to be formatted following the journal guidelines. Set your reference manager to the American Medical Association (AMA) citation style and make sure to include a PubMed ID at the end of each reference. You can search the PubMed IDs of articles at https://pubmed.ncbi.nlm.nih.gov/. It is also possible to copy your citation directly from the PubMed site provided it has been set to the AMA style (see references to this report for examples).

For articles without PMIDs, kindly include a DOI and ensure you verify your DOIs using https://www.doi.org/ to make sure they work.

37. For referenced websites, ensure to make as much effort as possible to get and reference the PDF version of the article (ie, in the absence of a PMID and DOI).

## Round 2 Review

### General Comments

The authors of the paper titled “COVID-19 Outcomes and Genomic Characterization of SARS-CoV-2 Isolated From Veterans in New England States: A Retrospective Analysis” have addressed all concerns raised close to full satisfaction. The paper is in much better shape now; however, there still are a few concerns worth noting. Kindly refer to the minor comments.

### Specific Comments

#### Minor Comments

1. Under “Study Design,” the second and third sentences should be moved to the “Study Setting” and the last sentence moved to “Ethical Considerations.” The justification for the study design initially recommended was to cite any studies on the topic that have used similar methods (if possible).

2. Tables 1 and 2 still need to be formatted according to the guidelines.

3. I still see the captions of figures appearing above the figures, contrary to the guidelines.

4. Kindly maintain the heading “Multimedia Appendix: Lineages of genomes” in the manuscript but remove the table and upload it in the online manuscript management system.

5. Ensure that all reported percentages in your manuscript are accompanied with the absolute values on which they were calculated, for instance, 25% (5/20) or (25%, 5/20).

## Abbreviations

AMA: American Medical Association
